# Crossfit analysis: a novel method to characterize the dynamics of induced plant responses

**DOI:** 10.1186/1471-2105-10-425

**Published:** 2009-12-16

**Authors:** Jeroen J Jansen, Nicole M van Dam, Huub CJ Hoefsloot, Age K Smilde

**Affiliations:** 1 Biosystems Data Analysis, Swammerdam Institute for Life Sciences, Faculty of Sciences, Universiteit van Amsterdam, Nieuwe Achtergracht 166, 1018 WV Amsterdam, The Netherlands; 2 Department of Multitrophic Interactions, Centre for Terrestrial Ecology, Netherlands Institute for Ecology, P.O. Box 40, 6666 ZG Heteren, The Netherlands

## Abstract

**Background:**

Many plant species show induced responses that protect them against exogenous attacks. These responses involve the production of many different bioactive compounds. Plant species belonging to the *Brassicaceae *family produce defensive glucosinolates, which may greatly influence their favorable nutritional properties for humans. Each responding compound may have its own dynamic profile and metabolic relationships with other compounds. The chemical background of the induced response is therefore highly complex and may therefore not reveal all the properties of the response in any single model.

**Results:**

This study therefore aims to describe the dynamics of the glucosinolate response, measured at three time points after induction in a feral *Brassica*, by a three-faceted approach, based on Principal Component Analysis. First the large-scale aspects of the response are described in a 'global model' and then each time-point in the experiment is individually described in 'local models' that focus on phenomena that occur at specific moments in time. Although each local model describes the variation among the plants at one time-point as well as possible, the response dynamics are lost. Therefore a novel method called the 'Crossfit' is described that links the local models of different time-points to each other.

**Conclusions:**

Each element of the described analysis approach reveals different aspects of the response. The crossfit shows that smaller dynamic changes may occur in the response that are overlooked by global models, as illustrated by the analysis of a metabolic profiling dataset of the same samples.

## Background

Most plant species are able to produce a wide range of defensive metabolites in response to attacks by pathogens or herbivores. This process is referred to as the induced plant response [1,2]. Due to their biological activity, induced plant chemicals form a rich source of plant natural products, such as insecticides and pharmaceuticals [3,4].

These plant responses are dynamic processes, in which different compounds may change in concentration at different times after the attack. The chemical identity of many inducible compounds is as yet unknown and the dynamics of known compounds may be elusive. Unknown compounds may be identified using a comprehensive analysis of the metabolic composition of these plants, referred to as 'metabolomics' [5-7]. Similar technological platforms may also be used in a more targeted analysis of specific compound classes, for example when the unknown dynamics of already known metabolites are of interest [8,9].

Metabolomic analyses provide information on a wide range of compounds, the concentrations and dynamics of which are mutually related through metabolic pathways. The interrelations between metabolites are therefore of considerable interest as well. The chemical data of such experiments is generally analysed using multivariate techniques that take these relationships into account [10-12]. The results of these multivariate methods consist of 'metabolic profiles', which are novel variables that are interpretable, canonical descriptors of all measured metabolites. These represent the most important 'modes of variation' [7], representing the variation between plants of different treatment groups or between plants in one treatment group.

Several multivariate methods have been used, or even specifically developed, for extracting such variation modes from time-resolved metabolomics experiments. Principal Component Analysis (PCA) is widely used to describe the data collected for all time-points simultaneously [13,14]. However in these PCA metabolic profiles many sources of information are confounded, which may seriously hamper the biological interpretation of these models [15]. Several other methods have been developed that take the experimental design into account: these lead to models that focus more on the experimental question that underlie the specific design. Examples of such methods applied in metabolomic analyses are Batch Processing [16], Partial Least Squares-Design of Experiments [17] and Geometric Trajectory (SMART) analysis [18]. These methods exclude metabolic variation that is not of interest to the experiment from the model and instead focus on dynamic and treatment-related variation.

Analysis of Variance-Simultaneous Component Analysis (ASCA) [19,20] specifically targets dynamical changes in treatment effects by imposing a model familiar from Analysis of Variance (ANOVA), on the data before fitting component models to each contribution in the linear model. This implies that all variation is disentangled into different factors and interactions imposed by the design, which can be independently interpreted and mutually compared. The metabolic profiles obtained from ASCA, as well as those from the other previously mentioned multivariate data analysis methods, describe the variation at all time-points into one model. Such 'global models' thereby focus on the most prominent modes of variation during the experiment. However, the response may also involve smaller modes of variation that take place only during a short time-span in the experiment. Such modes may be overlooked by a global model regardless of their potential interest to understanding the biological relevance of the response, such as minor changes in the levels of highly bioactive compounds.

Smaller modes of variation in the induced response may be revealed by focusing on limited time intervals during the experiment in a more 'local model'. The recently developed 'Piecewise Multivariate Modeling' [21] method fits independent metabolic profiles that describe differences between any two time-points in the experiment. However, it is not straightforward to model treatment effects using this method, since it only includes samples from treated individuals. It is therefore not straightforward to account for metabolic variation beyond experimental control in a Piecewise Multivariate Model, because the 'control' plants that may form the baseline for effects observed in the model cannot be included. The information provided by Piecewise Multivariate Models that describe treatment effects will be confounded by this uncontrolled variation.

To describe the metabolic response to perturbations in a poorly controlled environment such as a greenhouse, it is always essential to compare samples obtained from treated individuals--such as induced plants--to untreated control samples obtained at the same time-point. 'Local' multivariate models that describe the chemical variation between treated and control plants for each individual time-point may focus on the treatment effects in the metabolism. However, individual local models lack the common ground to compare between time-points, and therefore such a model cannot directly reveal the dynamic changes in an induced response.

To focus the local models on these dynamic changes, such common ground needs to be created. We propose to do this by 'crossfitting'. In the crossfit, the description of the chemical variation at a certain time-point by the 'local' metabolic profiles of other time-points in the study is established. Differences between these descriptions and the local models themselves then indicate the dynamic changes in the chemical background of the induced response, *i.e*. the emergence or disappearance of different modes of variation. This type of approach builds upon the work in psychometrics where comparisons were carried out between different factor analyses [22]. Our method is focussed on PCA and uses the model fit and the clustering of the scores as measures to compare the dynamic variation between study time-points.

To illustrate the analysis approach employing global models, local models and crossfit, a dataset describing the dynamic chemical variation of the induced plant response of feral cabbage plants (*Brassica oleracea*) was studied. Treatment of these plants with the plant hormone jasmonic acid (JA) leads to a response closely resembling that to chewing herbivore attacks [1]. It was applied either to the plant roots (*RJA*) or shoots (*SJA*) to create comparable responses to below- or aboveground herbivory, since both are known to differ chemically [23-25].

The most widely studied cabbage defence chemicals are the glucosinolates, because they possess both favourable and detrimental dietary properties and play key roles in the ecological interactions with herbivorous insects [3,26-28], among which antithyroid activity [29], chemoprotection against xenobiotics, as well as carcinogen activation [3,30]. Also, egg-laying in specialized herbivores and a response to JA have been associated with glucosinolates [24,25,31,32]. Glucosinolates may be analysed in a targeted fashion using High Performance Liquid Chromatography coupled to Ultraviolet detection (HPLC) [25].

Aside from the glucosinolates, many other metabolites may be involved in the induced plant response to JA. Therefore, the same samples were also analysed using an untargeted metabolomic analysis using Liquid Chromatography coupled to Mass Spectrometry (LC/MS) [33]. The dynamic behaviour of the induced plant response is analysed using the three-faceted approach for the profiling dataset and for the LC/MS data.

## Results and discussion

### Algorithm

#### The Global Analysis model

In this experiment we are mainly interested in the effect both treatments have on the phytochemical composition and in their dynamic aspects. Therefore, dynamic chemical changes that are common to all plants, e.g. those caused by environmental factors beyond experimental control, are not of interest to the experimental question. Inclusion of such variation modes into multivariate models can be avoided by separately centering the data of each individual time-point *i*, instead of that of all samples simultaneously as is commonly performed in PCA [34,35].

**Figure 1:**
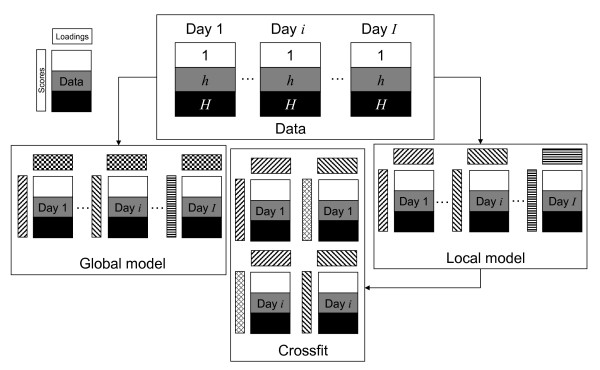
**Analysis scheme**. Depiction of the described analysis approach: The data of the treatment groups *h *nd days re arranged into *I *matrices. These can be analysed using a global model (left), where the same loadings are fitted for every day *i*. In the local model (right), different loadings are fitted for each day. The scores on these loadings are subsequently determined in the crossfit (center). The figure shows a crossfit between days 1 and *i*, but this procedure is possible between all days in the experiment.

Thereby the component model focuses more on the differences between treatment groups and the dynamic changes therein. When the resulting variation is modelled with one set of scores and loadings, the Simultaneous Component Analysis (SCA) model in equation (1) is obtained [36,37]. A schematic depiction of the global model, together with all other methods proposed in this analysis approach is given in Figure 1.(1)

Where **X**_*hi *_is the matrix containing all data of treatment group *h *at day *i *of dimensions (*N*_*hi *_× *M*), 1...*h*...*H *the index for plant treatment and 1...*i*...*I *the index for harvest day; *N*_*hi *_is the number of samples harvested at day *i *for treatment *h*, *M *is the number of measured variables;  is a length *N*_*hi *_column vector containing ones and vector **m**_*i *_of length *M *contains the mean value of each variable *m *for all samples obtained at day *i*; **T**_**g**,*hi *_is the score matrix with dimensions (*N*_*hi *_× *R*_**g**_) of the model fitted for day *i *and treatment *h *describing the variation among the plants; furthermore *R*_**g **_is the number of principal components, restricted as ; **P_g _**is the loading matrix of dimensions (*M *× *R*_**g**_) containing the glucosinolate profiles, where  is the matrix of dimensions (*N*_*hi *_× *M*) containing the model errors (i.e. residuals) of the day *i *and treatment *h *model.

A range of different SCA models is available, all varying in the constraints put on the score matrices ***T***_**g**, *hi*_. The least restricted SCA-P model may be obtained from a PCA on the concatenated data  that is first centered according to equation (1) [38]. Therefore, *R*_**g **_is equal for all days and can be evaluated using an external criterion, such as the scree plot [14]. Because all matrices **T**_**g**, *hi*_. are expressed on one basis **P_g_**, the scores **T**_**g**, *hi*_. obtained from this model can be compared between individuals (and between treatment groups), but also between time-points. Therefore they may be represented as time-series: we refer to this model as the 'global model'.

Because SCA models the variation of all days simultaneously, it gives much importance to relatively large modes of variation. Smaller modes, specifically those that occur only within a small time-frame in the experiment, may be overlooked in this model. A subsequent analysis that focuses more on such 'local' effects may therefore reveal additional information about smaller, yet possibly important models in the dynamic variation of the response.

#### The Local Analysis model

The PCA model fitted on the data of one harvest day *i*, fitted through least-squares, describes as much variation between plants on that harvest day as possible. Such a 'local model' is given in Equation (2).(2)

where **P**_*i *_is the PCA loading matrix for day *i *of dimensions (*M *× *R*_*i*_) and ; are the corresponding score matrices with dimensions (*N*_*hi *_× *R*_*i*_); *R*_*i *_is the number of principal components, restricted as ; **E**_*hi *_contains the model residuals, which are generally different from those in equation (1).

By interpreting the scores **T**_*hi*_, the variation in glucosinolate composition among all plants--and treatment groups--harvested on the same day *i *may be evaluated. However, unlike for **T**_**g**, *hi*_, these scores cannot be compared directly between days, because the data of each harvest day is expressed on a separate basis **P**_*i*_, which differs between harvest days. Such a comparison therefore requires simultaneous comparison between the corresponding loadings **P**_1_...**P**_*I *_to reveal differences between the chemical backgrounds underlying each day. Such a simultaneous comparison may however be too confounded and thereby remain too qualitative, specifically in the case of datasets with many variables, such as those obtained from metabolomic analyses. A more quantitative, model-based interrelation between models of different time-points may provide novel insights into evolving induced responses.

#### Crossfit of the local models

When two score matrices are being compared, they should preferably relate to the same loadings: to obtain such a comparison between different days from the local models from equation (2)--and thereby compare the treatment effects between days--the data matrices **X**_1*i*_...**X**_*Hi *_of plants harvested on day *i *may also be modelled by the local loadings of another day *j*. This leads to the 'crossfit model' in equation (3).(3)

where **T**_*ij *_of dimensions (*N*_*i *_× *R*_*j*_) contains the crossfit scores and *j *indicates the day at which the loadings were originally fit in equation (2); **E**_*h, ij *_contains the model residuals.

Generally the estimated mean vector **m**_*i *_in equation (2) is considered an integral part of the component model. However, in the crossfit it is specifically associated with the data that is being modelled, because modelling the data with the mean **m**_*j *_in equation (3) would lead to the inclusion of the difference between **m**_*i *_and **m**_*j *_in the crossfit model: this difference is the aforementioned dynamic variation between time-points *i *and *j *that is beyond control of the experiment (and equal for all plants): an undesired mode of variation.

The least-squares solution to obtaining the crossfit scores **T**_*ij *_can be obtained by projecting the data **X**_*i *_on the loadings **P**_*j*_. Because the loading matrices are all orthogonal, this projection is given by , which implies that in equation (3),  is minimised for given **P**_*j*_. The orthogonality of the loadings of different components to each other and to the residuals  then ensures that the fit of **T**_*h*, *ij *_to **X**_*i *_can be calculated analogously to that of the PCA scores. Note that for day, *i *= *j *equation (3) generalises to the local PCA model in equation (2), such that **T**_*h*,*ii *_= **T**_*hi*_.

Because the local PCA models minimise,  the percentage of variation explained in a crossfit model where the data of day *i *is projected on the model of day *j *will always be equal or lower than that of the PCA model. The percentage for a model where the data of day *j *is projected on the day *i *model is not restricted with respect to the local PCA fit for day *i*. Note these constraints hold only for the cumulative percentage of variation explained by the entire model and not necessarily *per *principal component. Furthermore, because we perform the crossfitting on all plants harvested at the same day simultaneously, the fit is aggregated here for all treatments even though this is not strictly necessary.

Crossfitting by equation (3) does not necessarily require *R*_1 _=...= *R*_*I*_, although the maximum number of principal components of each local model is restricted: to crossfit among all days, the maximum number of principal components for all local models is restricted to . The size, separation and shape of clusters in **T**_1,*ij*_...**T**_*H*,*ij *_may be interpreted in much the same way as in the original PCA scores.

The crossfit may provide dynamic information using both the amount of explained variation, as well as the clustering in the crossfitted scores. Large differences between the percentage of explained variation by crossfitted scores compared to principal component scores indicate the presence of dynamic changes in the chemical profiles underlying the induced response: additional, or at least different modes of variation are present at both time-points. When data of a specific day is crossfitted on a model describing additional modes of variation compared to the local PCA model, no considerable loss in the fit of the data should occur: these additional parts of information in the model will be redundant for describing the variation in the data but will not lead to a poorer fit.

Further insight into the dynamics of the individual modes of variation may be obtained by observing clustering in the local PCA scores **T**_*hj *_and in the crossfitted scores **T**_*h,ij *_on the loadings **P**_*j*_. Analogously, the crossfitted scores **T**_*h*,*ij *_may be compared to the PCA scores of that day **T**_*hi*_. However, in this latter comparison it should be kept in mind that both scores pertain to the same samples, but are expressed on different bases.

## Implementation

**Figure 2:**
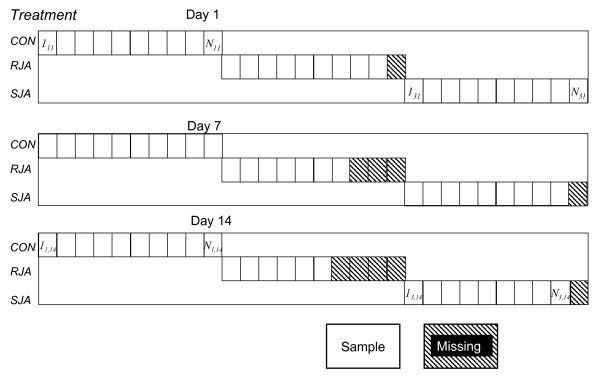
**Experimental design**. The design comprises 3 harvest days and 3 treatments; 10 plants were harvested *per *treatment *per *day. The destructive harvest implies that different plants are chemically analysed for each harvest day; several plants were lost in sample preparation, indicated by the diagonal stripes. In the figure, *CON *has been indexed by 1, *RJA *by 2 and *SJA *by 3.

The induction experiment provided plant samples that received one of three treatments and were harvested at one of three days. The preparation and chemical analysis of the plants is described in the methods section. The glucosinolate composition of several plants could not be determined due to an error in the sample preparation: these were removed from the glucosinolate and metabolomic data. The experimental design, indicating the number of remaining replicates per harvest time/treatment combination is indicated in Figure 2. The plant samples were analysed using two platforms. First the targeted analysis of glucosinolates will be used to show how insight into the different modes of variation may be connected between the global model, the local models and the crossfit. After that, the LC/MS-based metabolomic analyses are used to show how the crossfit model may be used to generate novel insights.

### The Global Model

**Figure 3:**
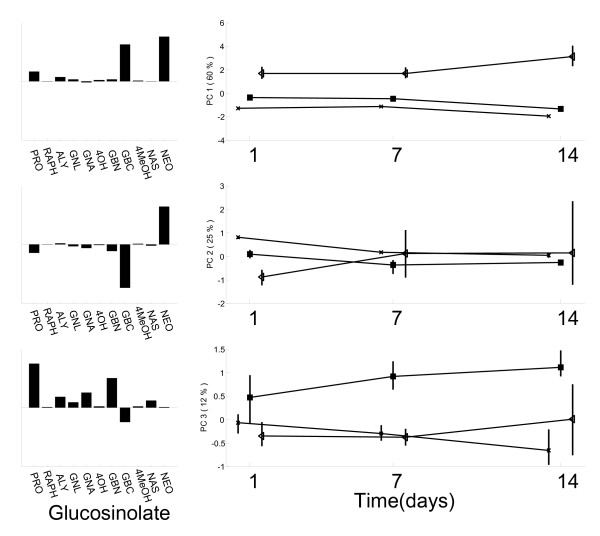
**Global SCA model of the glucosinolate data**. Loadings (left) and Scores (right) for principal components 1, 2 and 3 (top to bottom). *CON *plants have been indicated by crosses, *RJA *plants by squares and *SJA *plants by triangles. The vertical lines indicate the 95% confidence interval of the scores. The percentages of variation explained by each component are in the figures.

The global model required three principal components to describe 97% of the total variation in the data (Figure 3). This model revealed three distinct modes of variation: the mode with which most variation is associated, therefore described by the first principal component, relates to the elevated concentrations of neoglucobrassicin and glucobrassicin (NEO and GBC): a list of glucosinolates identified in these plants, together with their common and chemical names is given in Table 1. This elevation is larger for *SJA *plants than for *RJA *plants and is present throughout the experiment.

**Table 1:** List of used glucosinolate abbreviations, their common names and their chemical names as given by Fahey *et al*. [47]

Abbreviation	Common Name	Chemical name
PRO	Progoitrin	2(R)-2-Hydroxy-3-butenyl

RAPH	Glucoraphanin	4-(Methylsulfinyl)butyl

ALY	Glucoalyssin	5-(Methylsulphinyl)pentyl

GNL	Gluconapoleiferin	2-Hydroxy-4-pentenyl

GNA	Gluconapin	3-Butenyl

4OH	4-Hydroxyglucobrassicin	4-Hydroxyindol-3-ylmethyl

GBN	Glucobrassicanapin	4-Pentenyl

GBC	Glucobrassicin	Indol-3-ylmethyl

4MeOH	4-Methoxyglucobrassicin	4-Methoxyindol-3-ylmethyl

NAS	Gluconasturtiin	2-Phenylethyl

NEO	Neoglucobrassicin	1-Methoxyindol-3-ylmethyl

The second mode of variation comprises a negative correlation between the same glucosinolates and reveals itself in two ways. On the second component we see a clear difference between the three treatment groups for day 1. The difference between the three groups on day 1 is not fully described by the first component: the remainder of this variation can be described by a negative relation among NEO and GBC and is described by the second component of the global model. This 'contrast' between NEO and GBC is referred to as mode of variation 2A. On the other two days the negative correlation between both glucosinolates relates to a large spread among *SJA *plants. This mode of variation, with the same chemical background as mode 2A, is referred to as mode of variation 2B.

The third mode of variation that can be identified from the global model is an elevation in the concentrations of several biochemically related glucosinolates: progoitrin, glucobrassicanapin and gluconapin (PRO, GBN and GNA), increasing in concentration between 1 and 7 days after treatment. The three identified modes of variation are summarized in Table 2 and will be used for further interpretation of the local models and the crossfit of the glucosinolates.

**Table 2:** Modes of variation identified in the glucosinolate data

#	Variation among plants	Chemical background	Time-points
1	Increased concentrations in *SJA *and to a lesser extent in *RJA *plants	Positive correlation among NEO and GBC	1-14

2	A. Contrast compensating for relative differences in glucosinolate levels between day 1 and the other days	Negative correlation among NEO and GBC	1 (*SJA*) 1-14 (*RJA*)
			
	B. Increased variation among *SJA *plants compared to *CON *and *RJA *plants		7-14

3	Increased concentrations in *RJA *plants compared to *CON *plants	Positive correlations among PRO, GBN and GNA	7-14
		
4	Increased variation among *RJA *plants		1

These model results show that the induced response depends on where on the plant JA was administered, which was already known [25]. However, the global model also shows that both responses are dynamic and that thereby the treatment-induced glucosinolate differences between the treatment groups also change in time.

### The Local models

The local models require two principal components on day 1 and three on days 7 and 14, following from the percentage of explained variation of each component (Table 3).

**Table 3:** % of variation explained by each component in a local PCA model

	Day		
**PC**	**1**	**7**	**14**

1	83	58	64

2	13	28	23

3	2	12	11

4	1	1	1

The number of components selected for each model is underlined

**Figure 4:**
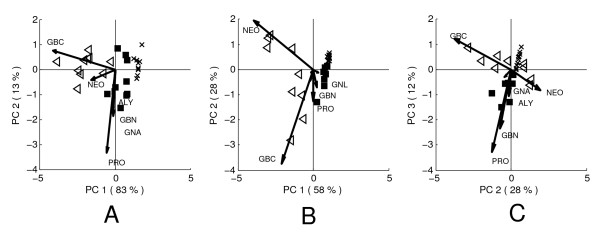
**Biplots of the local PCA models of the glucosinolate data**. A) Day 1, PC 1 vs. 2, B) Day 7, PC 1 vs. 2, C) Day 7 PC 2 vs. 3; *CON *plants have been indicated by crosses, *RJA *plants by filled squares and *SJA *plants by open triangles. The arrows indicate the loadings and the loading labels indicate the corresponding glucosinolate species. The figure order corresponds to the modes of variation described by the global model components.

The PCA model results (Figure 4) are given as 'biplots' [39]. The scores in such plots may be interpreted analogous to ordinary PCA score clusters. The loadings however, are indicated by arrows in the same plot, which gives insight to the relationships between treatments and individual glucosinolates. When a loading points in the same direction as a difference between two score clusters, the concentration of that glucosinolate is higher in the treatment group that the loading points towards. The relative importance of each glucosinolate in a mode of variation is indicated by its (relative) length. Furthermore, two glucosinolates for which the loadings point in the same direction have positively correlated concentrations. Likewise, opposite loading directions indicate negative correlations.

Mode of variation 1 is expressed on the first principal component of the day 1 local model (Figure 4A). The loadings of NEO and GBC are most associated with this mode of variation in the figure. However, comparison of the day 1 local model to the first component of the global model (Figure 3) shows that the relative contributions of both glucosinolates differ between both models. Mode of variation 2A that was interpreted from the second global principal component is therefore also included in this local model, leading to the more parsimonious description of the day 1 variation by the local model into two rather than the three global model components. The model shows the difference between *SJA *and *CON *plants is larger but lies in the same direction as the difference between *RJA *and *CON *plants: the same relative contribution of NEO and GBC underlies both induced responses.

The second principal component of the local day 1 model describes an increased variation among *RJA *plants on PRO, GNA and GBN compared to *CON *plants. This mode of variation is relatively small and was therefore not identified in the global model, but is only found here as a fourth mode of variation (Table 2). Because the loadings on the second component of the day 1 local model are similar to those on the third component of the global model, both modes may be chemically associated.

The day 7 local model shows that the glucosinolate profiles associated with both induced responses have changed compared to day 1. The concentrations of NEO and GBC are elevated in both *RJA *and in *SJA *plants, compared to *CON *plants. Unlike at day 1, the relative contributions of NEO and GBC differ between both responses. The effects of both treatments on the levels of NEO and GBC can therefore not be described by the same principal component, but requires two principal components (Figure 4B). In addition, the cluster size of the *SJA *plants is much larger than that of the *RJA *and *CON *plants, specifically on the second component. The opposite direction of the NEO and GBC loadings on this component indicates this corresponds to the negative correlation identified in the global model as mode of variation 2B. Mode of variation 3, mainly involving PRO and GBN, is revealed on the third principal component of the day 7 model (Figure 4C).

The local models confirm that the *JA*-induced profiles indeed strongly differ between days. Furthermore they show that smaller modes of variation that remain elusive in the global model may be extracted from models that focus on individual days.

### Crossfit analysis of the local models

The percentage of explained variation from the crossfit (Table 4) reveals that the day 1 model fits the day 7 and 14 data poorly, which indicates the glucosinolate profiles describing the induced response show considerable dynamic change. The lower number of components chosen for the day 1 local model does not sufficiently explain this loss, because the third principal components of both local models explain considerably less variation (12 and 11% respectively, Table 3). The complementary crossfit shows that the day 7 and 14 models explain the variation between the plants harvested at day 1 well (i.e. 95 and 96% respectively), indicating the modes of variation on day 1 remain present at these days. In the crossfit between the day 7 and day 14 local models hardly any loss of fit occurs, indicating both glucosinolate profiles are exchangeable.

**Table 4:** Crossfit analysis of data collected and modelled using PCA after 1, 7 and 14 days

		Model Day		
		
		1	7	14
Data	1	95	95	96
	
Day	7	55	98	97
	
	14	55	97	97

Underlined numbers indicate the PCA model fit

**Figure 5:**
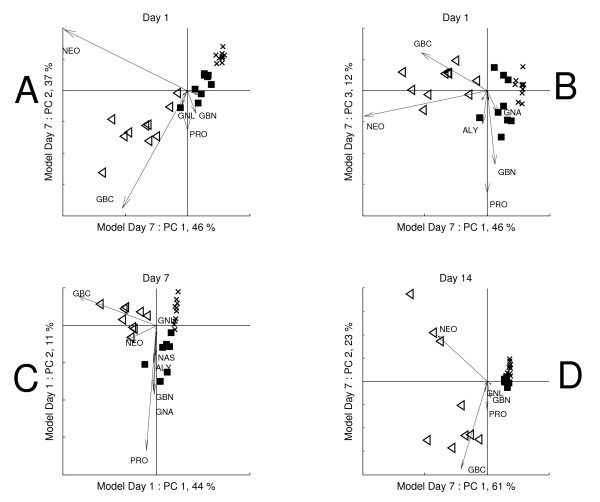
**Crossfitted scores of the glucosinolate data**. Day 1 data crossfitted on day 7 principal components for A) PC 1 vs, 2 and B) PC 1 vs. 3; C) Day 7 data crossfitted on day 1 model, D) Day 14 data crossfitted on day 7 model; *CON *plants are indicated by crosses, *RJA *plants by squares and *SJA *plants by triangles. The arrows indicate the loadings of each local model.

The crossfit of the day 1 data on the day 7 model confirms that mode of variation 2B is absent on day 1: the difference between the three treatment groups can be described by one dimension in Figure 5A, although this dimension does not correspond to a single component like it does in the day 1 local model. The large variation among *SJA *plants on the second component observed in the day 7 local model (Figure 4B and 4C) is absent in the day 1 crossfitted scores.

Also mode of variation 3 is absent in the day 1 data (Figure 5B). However, instead of this mode, the mode of variation 4 emerges on the third component of the day 7 local model (Figure 5B). This gives further evidence that the third and fourth modes of variation may be chemically related. Comparison of Figure 5B to the local day 1 model (Figure 4A) shows the scores in both figures are very similar: this supports the combined observations that the variation on day 7 corresponds to the variation on day 1, in which an additional mode of variation 2B has emerged and where mode 3 has replaced mode 4.

The crossfit of the day 7 data on the day 1 model provides the complementary evidence of the previous observations (Figure 5C). Mode of variation 2B is absent in these scores, confirming the day 1 model does not describe it. Furthermore, relations between modes of variation 3 and 4 are further confirmed because the second component describes mode of variation 4 (Figure 4A). Finally, the crossfit scores of the day 14 data on the day 7 model (Figure 5) closely resemble those of the day 7 PCA model (Figure 4B), which confirms both models are exchangeable because they describe the same glucosinolate chemistry.

The crossfit allows for a comparison between the chemistry underlying the response-related variation observed on different days: chemical relations between different modes of variation can be revealed and absence or presence of different modes of variation on each day can be confirmed. The crossfit proves to be useful interpreting the results of the glucosinolate analysis, because it may be used to connect the observations in the local models (Figure 4) to each other and thereby facilitates easier connection to the global model (Figure 3).

The three-faceted approach employed here reveals that the glucosinolate arsenal presented by the plant to its surroundings is rather intricate, as indicated by the analysis of this dynamic study. The global analysis reveals the induced response of *B. oleracea *to *JA *is very long lasting: the glucosinolate response of the related oilseed rape (*B. rapa*) upon the same treatment with jasmonic acid lasted for between 7 and 14 days, showing that the response of the feral plants used in this study lasts longer [31]. The first mode of variation has been observed in these feral plants before, both for *RJA *and *SJA*. However, the second mode of variation (i.e. the negative correlation between NEO and GBC) was hitherto unknown and could only be found using a multivariate approach. The dynamics of this variation mode could be revealed by the local models and the absence of this mode after 1 day could be confirmed using the crossfit analysis. As the enzyme converting GBC into NEO has not been isolated and identified yet, this information may be used to harvest induced plants at a time point when the enzyme is highly active and/or the gene coding for the enzyme is highly expressed. The third mode of variation was also hitherto unknown and although the fourth mode was observed before, its late response relative to that of NEO and GBC was a novel finding from the three-faceted approach employed in this dynamic experiment [25]. The observed modes of variation comply with the biosynthetic origin of the glucosinolates: NEO and GBC are both indole glucosinolates, derived from the amino acid tryptophan. The glucosinolates PRO, GBN and GNA are all three derived from the amino acid methionine: they are closely biosynthetically related [40]. Also GNL shares this biosynthetic relation, but the relatively low levels of this compound may have decreased its influence on the generated multivariate models.

### The three-faceted approach applied to metabolomics data

The crossfit may also provide information that cannot be obtained from the global and local models, which is shown by the analysis of the metabolomic measurements of the same samples. To focus on the added value of the crossfit we will limit ourselves to days 7 and 14.

**Figure 6:**
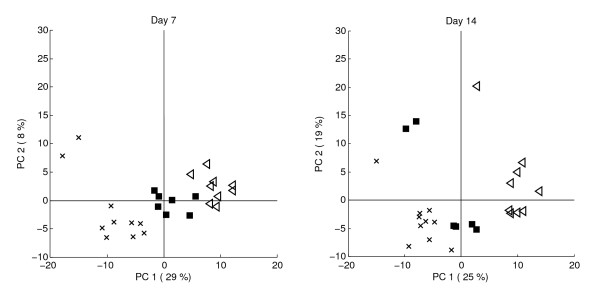
**Global model scores of LC/MS data**. Global models of Day 7 (left) and Day 14 (right); *CON *plants are indicated by crosses, *RJA *plants by squares and *SJA *plants by triangles.

The global model fitted on days 7 and 14 shows that the three treatment groups can be chemically distinguished by the same metabolomic profile, consisting of two principal components (Figure 6). Furthermore, the direction of the difference between the *CON *and *SJA *plants is similar to that between *CON *and *RJA*: the response to both treatments is chemically similar but different in magnitude. Also both days exhibit the same direction, which indicates that judging from this model the response is not dynamic and not treatment-specific.

**Figure 7:**
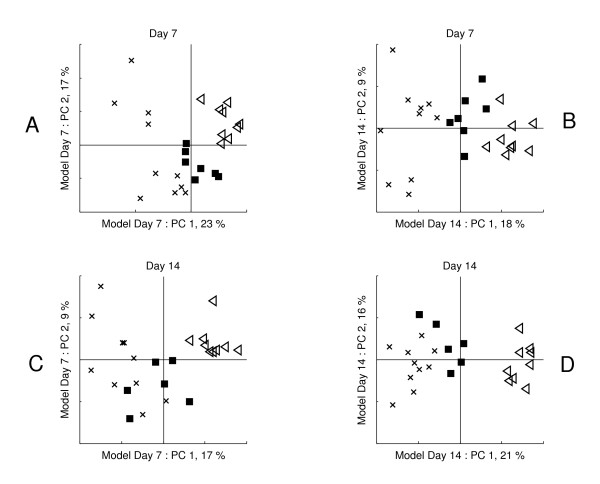
**Local and Crossfitted scores of LC/MS data**. Scores of A) the day 7 model, B) the Day 7 data crossfitted on day 14 model, C) the Day 14 data crossfitted on day 7 model. D) the local day 14 model; *CON *plants are indicated by crosses, *RJA *plants by squares and *SJA *plants by triangles.

The local models show that the difference between the treatment groups lies in a different direction for the day 7 than for the day 14 model (Figure 7A and 7B). However, this does not imply that the chemical background differs between both days, since both local models lack the aforementioned common ground.

The crossfit of the day 7 data on the day 14 model shows that the differences between the treatment groups on day 7 can be explained well by the day 14 model (Figure 7C). However, the crossfit of the day 14 data on the day 7 model leads to an overlap between the clusters of the *CON *and *RJA *plants. Therefore the chemical background of the differences between both plant groups differs between day 7 and day 14 (Figure 7D). These differences can be revealed by a comparison of the loadings between both local models, which goes beyond the scope of this paper.

This shows that the focus of global models on the largest modes of variation throughout the experiment (i.e. here the modes that appear both on day 7 and 14) may lead to the conclusion that there is no dynamic variation in the responses. However, the local models contain information that shows the response is in fact dynamic. The crossfit between the local models is then essential to reveal such dynamic variation from the different local models. Thereby crossfit may also provide novel information that could not be obtained from a global model, or from the local models themselves.

The proposed approach forms a generic framework, by which the effect of several treatments (i.e. *RJA *and *SJA *in this experiment) can be compared among different measurement occasions. In this experiment different harvest times were compared, but this may be replaced by any other factor (e.g. temperature, nutritional regime). Central to the approach is that the same variables have been measured for each treatment and measurement occasion.

An essential aspect to take into account while comparing local models and interpreting the crossfit is that the basis of each local model individually describes the largest variation at its specific measurement occasion. This means the position of each score relative to the other scores may be compared between local model loadings, but the absolute position of the scores may not. The absolute positions of the local model and crossfit scores expressed on different bases may be made more comparable by performing an orthogonal rotation of the basis [41].

## Conclusions

The crossfit analysis that we introduced here revealed that inducing *B. oleracea *plants leads to considerable response in plant chemistry. This response is highly dynamic, such that observation of and comparison between different time-points is important for the understanding of the response. Therefore three interrelated methods were used to describe the chemical variation. Although all three were based on Principal Component Analysis, their different focus on describing the chemical variation put forward different aspects of the response.

The glucosinolate analyses showed that the global model, that describes all days simultaneously, provided easy to interpret time-profiles. Local models, describing only one day at a time, added information about smaller chemical changes involved in the response. The novel 'crossfit' analysis was developed because local models of different days cannot be directly compared to each other. The results of the glucosinolate analyses showed that the crossfit may prove insightful in linking observations in different local models to each other and to specific 'modes of variation' observed in the global models. Furthermore the results of the metabolomic analyses revealed that the crossfit may also be essential in extracting dynamic variation from the local models that could not be obtained from the global models.

The range of applicability of the three-faceted approach may reach beyond examining dynamics: this method may very well be used to gain insight into many more comparable multi-factorial problems: for example, a growing field of interest is that of multi-compartmental interactions within the same responding organism, for which this method can be directly applied [42]. Also the comparison of information about the same samples from different analysis platforms (such as the glucosinolate and metabolomic analyses used here) may be examined using an analogous approach [8]. The three-faceted approach is also not limited to PCA, but many more unsupervised and supervised multivariate methods may be used.

The combination of global and local models and of the crossfit into one three-faceted approach provides a bioinformatic tool that is easy to implement, which may considerably extend the insight into a wide range of complex multi-factorial biological systems.

## Methods

### Plant rearing, treatment and harvest

Seeds of *B. oleracea *L., mass collected in 2000 from natural populations near Heteren, The Netherlands, were germinated on glass beads in water. Seven days after germination, seedlings were transferred to 1.3-l pots containing fine river sand and covered with aluminium foil to reduce evaporation. The plants were placed in a glasshouse (21°C during daytime and 16°C at night). Light conditions were ambient, supplied with sodium lamps to maintain the minimum PAR at 225 μmoles *m*^-2 ^*s*^-1 ^at least 16 hours day^-1^.

We supplied the plants with half-strength Hoagland nutrient solution of which the KH_2_PO_4 _concentration was doubled; otherwise *B. oleracea *suffers from phosphorus deficiency in plain sand. Water content in the pots was maintained at 15%, by weighing five pots every 2-3 days and adding sufficient nutrient solution. Every week the water content of each individual plant was corrected by adding water, such that each plant received the same amount of nutrients.

Thirty days after transplantation of the seedlings, 120 plants of equal size and stature were selected and randomly distributed over the three treatment groups. Each plant received either a root (*RJA*) a shoot (*SJA*) or a control (*CON*) treatment with JA, as described before [25]. After 1, 7 and 14 days post-treatment, 10 plants were randomly chosen from every treatment group. These were harvested, consisting of cutting the plant shoot above the root knot, flash-freezing it in liquid N_2_, freeze-drying (Labconco, http://www.labconco.com/) and subsequent grinding in a ball mill (Retsch, http://www.retsch.com/).

### Chemical analysis

Glucosinolates were extracted with boiling 70% methanol solution, desulphatased with arylsulphatase (Sigma, St. Louis, IL, USA) on a DEAE-Sephadex A 25 column (EC, 1990) and separated on a reversed phase C-18 column on HPLC with an acetonitrile-water gradient [43]. Detection was performed with a single wavelength detector set to 226 nm. Sinigrin (sinigrin monohydrate, ACROS, New Jersey, USA) was used as an external standard. We used the correction factors for detection at 226 nm to calculate the concentrations of the different modes of glucosinolates in both plant species [44]. Triplicate analysis of the same plant sample revealed that glucosinolate concentrations could be assessed with an accuracy ranging from 0.04% (ALY) to 2% (NAS) of the average level. This analysis revealed 11 different glucosinolates in these plants.

The metabolomic analyses of shoot samples (48-52 mg aliquots) from the same plants was performed with reversed-phase Liquid Chromatography coupled to Quadrupole Time-Of-Flight Mass Spectrometry (LC-QTOF-MS) using the protocol for freeze-dried material described by De Vos *et al*. [33]. This protocol also discusses the analytical reproducibility of this method for these samples. The obtained spectra were preprocessed using MetAlign v.220805 and the peaks belonging to the same metabolite were clustered using the approach described by Tikunov *et al*. [45,46], which lead to 675 chemical descriptors of the plant metabolism.

## Online Availability

A MATLAB program for the crossfit is available *via *http://www.bdagroup.nl

## Authors' contributions

JJJ wrote the paper, designed the experiment, collected the data and developed the method; NMvD designed the experiment, collected the data, edited the paper; HCJH developed the method and wrote the paper and AKS developed the method and wrote the paper. All authors have read and approved the final manuscript.
